# Supplementary material for: Netcombin: An algorithm for constructing optimal phylogenetic network from rooted triplets
Source: PLoS One. 2020 Sep 18;15(9):e0227842. doi: 10.1371/journal.pone.0227842 (PMC7500971; doi:10.1371/journal.pone.0227842)
Supplement: S4 File — (ZIP) [file pone.0227842.s004.zip › SequenceData-Triplet/sequenceData-10-20-30-0-Triplet/length 100, numseq 40, rate 10^-7/9 seq.pdf]

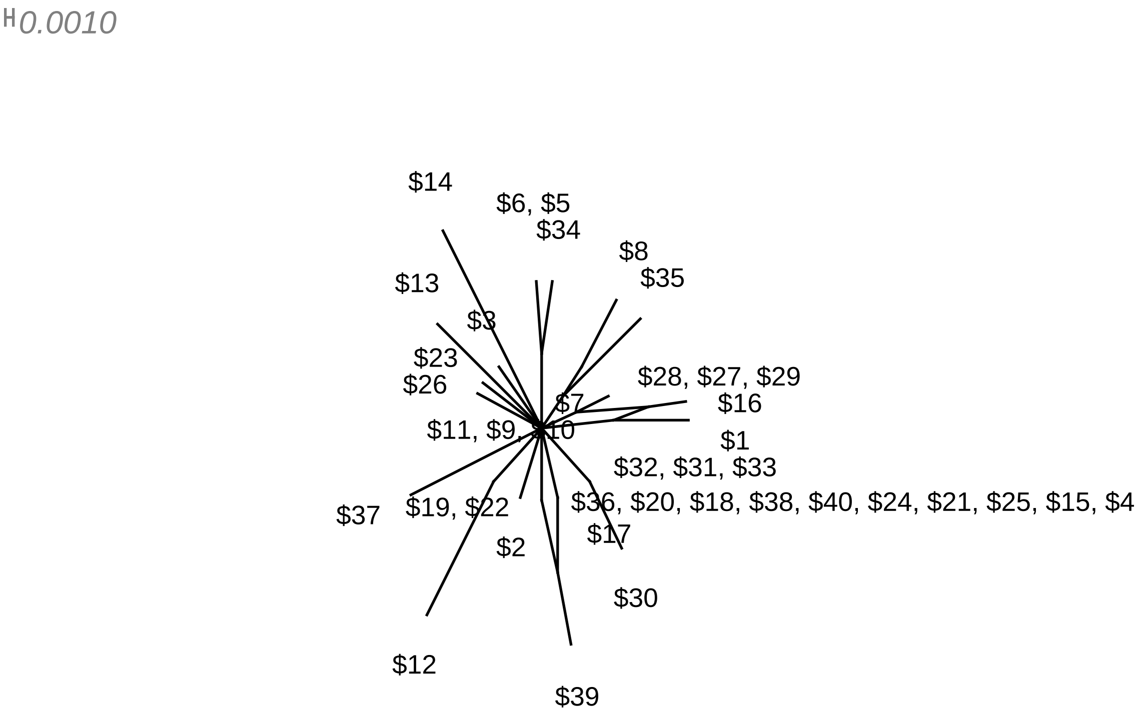

0.0010
$14
$6, $5
$34
$8
$35
$13
$3
$23
$26
$28, $27, $29
$16
$7
$11, $9, $10
$1
$32, $31, $33
$36, $20, $18, $38, $40, $24, $21, $25, $15, $4
$37
$19, $22
$17
$2
$30
$12
$39
